# Hypothesis on the Role of Cryptochromes in Inflammation and Subarachnoid Hemorrhage Outcome

**DOI:** 10.3389/fneur.2017.00637

**Published:** 2017-11-28

**Authors:** Adriano Barreto Nogueira, Ariel Barreto Nogueira, José Carlos Esteves Veiga, Manoel Jacobsen Teixeira

**Affiliations:** ^1^Division of Neurosurgery Clinic, Hospital das Clinicas, Faculty of Medicine, University of Sao Paulo, Sao Paulo, Brazil; ^2^Discipline of Neurosurgery, Santa Casa Faculty of Medical Sciences, Department of Surgery, Sao Paulo, Brazil; ^3^Faculty of Medicine, Department of Radiology, Hospital das Clinicas, University of Sao Paulo, Sao Paulo, Brazil; ^4^Faculty of Medicine, Department of Neurology, University of Sao Paulo, Sao Paulo, Brazil

**Keywords:** cryptochromes, subarachnoid hemorrhage, inflammation, neurogenesis, circadian rhythm, intracranial pressure prediction, therapeutic hypothermia, targeted temperature management

## Abstract

We have recently found that the temperature variability (TV) in the day–night cycle may predict the mean intracranial pressure in the following 24 h (ICP_24_) in subarachnoid hemorrhage (SAH) patients under multimodality monitoring, sedation, and hypothermia (<35°C). Specifically, we found that ICP_24_ = 6 (4 − TV) mmHg. TV is the ratio between the coefficient of variation of temperature during the nocturnal and the preceding diurnal periods. This result suggests that the circadian clock reflects brain plasticity mechanisms and its malfunctioning leads to deterioration of the neurologic status. The sleep–wake cycle is absent in these patients and their circadian clock can function properly only by environment light-independent mechanisms. One mechanism involves the circadian clock proteins named cryptochromes (CRYs). CRYs are highly preserved and widespread in the evolutionary tree, are expressed in different cell types in humans [type II CRYs, in two forms: human cryptochrome 1 and 2 (hCRY1 and hCRY2)], and in certain species, respond to blue light and play role in magnetoreception. Interestingly, SAH outcome seems to correlate with inflammation, and CRYs decrease inflammatory activity. Our hypothesis derived from these observations is that CRYs modulate the circadian oscillation of temperature even during therapeutic hypothermia and improve outcome in SAH through decrease in inflammation. A strategy to test this hypothesis is to measure periodically during the acute phase of high-grade SAH the level of CRYs in cerebrospinal fluid (CSF) and circulating white blood cells, and to correlate these levels with outcome, TV, ICP_24_, and pro- and anti-inflammatory markers in CSF and blood. If this hypothesis is true, the development of therapies targeting inflammation in SAH could take advantage of cryptochrome properties. It has been shown that blue light phototherapy increases the expression of CRYs in blood mononuclear cells in jaundiced neonates. Likewise, visual stimulus with flashing light improves Alzheimer’s disease features in experimental model and there is a prominent expression of CRYs in the retina. Remarkably, recent evidence showed that hCRY2 responds to electromagnetic fields, which could be one elusive mechanism of action of transcranial magnetic stimulation and a reason for its use in SAH.

## Introduction

Our previous work indicates that circadian rhythms are a primary factor to predict brain injury (1–6). First, we showed that the anterior hypothalamus displays a prominent expression of neurogenesis-related markers in adult humans (5, 6). The functions of the anterior hypothalamus include circadian rhythm control and thermoregulation. Because of these features, we suspected that the monitoring of circadian oscillation of temperature could measure putative brain regeneration and plasticity mechanisms and, therefore, anticipate the intensity of brain injury. Following this rationale, we discovered that the ratio between the mean temperature during the sleep and the preceding wake period correlates with the likelihood of seizure occurrence in the following 24 h in epileptic patients (1, 3). We have recently showed that this concept works even with high-grade subarachnoid hemorrhage (SAH) patients under sedation and therapeutic hypothermia induced by intravascular catheter (2). In this case, the ratio between the coefficient of variation of temperature during the nocturnal and the preceding diurnal periods correlates with the mean intracranial pressure in the following 24 h (ICP_24_) (2). Collectively, these findings open the possibility to reduce brain injury through circadian rhythm modulation.

Circadian rhythms may be modulated by interfering on the level of the clock proteins cryptochromes (CRYs). CRYs are evolutionarily old flavoproteins expressed in two types, in species ranging from plants to humans (7). Mammals display ubiquitous expression of type II CRYs (8, 9). In humans, there are two forms of type II CRYs, named human CRY1 (hCRY1) and CRY2 (hCRY2) (8, 9). CRYs are highly expressed in the suprachiasmatic nucleus (10). The suprachiasmatic nucleus is the hypothalamus structure that orchestrates the circadian rhythms by the central nervous system. CRYs also interfere in the other mechanism of circadian rhythm control (peripheral mechanism), which includes immune system cells (7). Importantly, hCRY levels can be potentially modulated by blue light (11) and electromagnetic field (8), which are currently used in clinical practice.

## Hypothesis

We hypothesize that the ratio between the level of expression of hCRYs during the nocturnal and the preceding diurnal periods (CRY_n/d_) correlates with inflammation and predicts neurologic signs in the following day–night cycle and long-term outcome in SAH.

## Rationale of the Hypothesis

### Adult Human Neurogenesis

The discovery of adult mammalian neurogenesis opened a novel perspective on brain plasticity and neuroregeneration therapies (4–6). The current prevailing concept that the adult mammalian brain harbors two primary neurogenic niches in the subgranular zone of the dentate gyrus and in the subventricular zone replaced the long-lasting dogma of no-new neurons after birth (4–6). Adult human neurogenesis is controversial, but we have previously shown that the adult human brain harbors what we referred to as potential neurogenic system (4–6). The potential neurogenic system was revealed by the detection of expression of neural stem cell markers in the circumventricular organs (4–6). The circumventricular organs are the brain structures located principally in the hypothalamus that display no blood–brain barrier. Moreover, brain structures adjacent to the circumventricular organs, which form part of the hypothalamic and limbic system circuits, express the immature or newly formed neuron marker doublecortin (DCX) (5, 6). We interpreted these results as a potential constitutive mechanism of neurogenesis that could participate in the maintenance of certain brain circuits and in their regeneration after injury. Next, we pursued a method to measure the effects of this potential plasticity mechanism in clinical practice.

### Brain Injury Prediction through Circadian Rhythm Monitoring

#### Presence of Sleep–Wake Cycle

Because the hypothalamus displays robust expression of neurogenesis-related markers and modulates circadian rhythms and temperature, we suspected that these functions parallel endogenous brain plasticity. Indeed, alterations in the hypothalamus-related functions anticipate worsening in neurologic status, maybe partially due to alterations in endogenous neurogenesis mechanisms. In practice, we found that the ratio between the mean skin temperature during sleep and the preceding wake period (T_s/w_) correlates with the likelihood of seizure occurrence in the following 24 h in epileptic patients (1, 3). This method is non-invasive and involves the use of wristband with sensors of vital signs (1, 3). Importantly, skin temperature has been shown to be a reliable circadian rhythm marker (1, 3).

#### Absence of Sleep–Wake Cycle

A stronger indication that the circadian rhythm is a primary factor to predict brain injury is being unveiled by our ongoing research showing that the pattern of oscillation of core body temperature in the day–night cycle in high-grade SAH patients correlates with further intracranial pressure (2). The patients analyzed in this study underwent sedation and targeted temperature management (33–33.9°C) using intravascular catheter. Circadian rhythm correlated with further neurologic signs even in this situation of decreased level of consciousness, lack of sleep–wake cycle, and strict temperature control. Specifically, the ratio between the coefficient of variation of the nocturnal (starting at 18:00) and the preceding diurnal periods (starting at 6:00) [temperature variability (TV)] correlated with ICP_24_ (*p* < 0.001). The formula derived from regression analysis is ICP_24_ = 6 (4 − TV) mmHg (2).

The findings of our line of research indicate the existence of a light-independent mechanism of circadian rhythm that participates in brain plasticity (9, 12). The discovery of a factor involved in this mechanism could lead to the development of a circadian rhythm-guided therapy to prevent brain injury.

### Cryptochrome As Potential Prediction Factor in SAH

Cryptochromes display circadian expression in the suprachiasmatic nucleus (10) and in peripheral tissues (7) such as white blood cells (11) through light-independent (7, 9) and light-dependent mechanisms (8, 10, 12).

The expression of hCRYs decreases during the day and increases during the night (13). Insufficient increase in hCRYs during the night correlates with higher levels of inflammation in the following morning, as has already been demonstrated in rheumatoid arthritis patients (13). In SAH patients, inflammation seems to lead to poor outcome (4).

**Figure 1:**
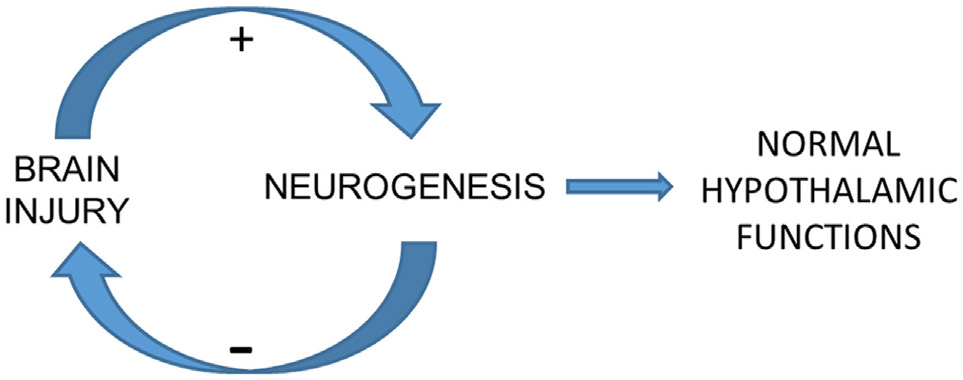
Elusive mechanism of the correlation between cryptochrome expression, temperature variability (TV), and further intracranial pressure (ICP). The potential neurogenic system that we described recently may be a background mechanism of brain plasticity whose core is located in the hypothalamus. This system may be modulated by the redox state of brain cells, which is remarkably altered in brain injury. An effective injury-induced neurogenesis would restore hypothalamus-related functions such as circadian rhythms and next autonomic functions related to cerebral autoregulation, contributing to ICP control. TV seems to be a marker of circadian rhythm even in comatose and sedated patients undergoing hypothermia. This condition implies the role of a light-independent mechanism of circadian rhythm control, which may be played by hCRY expression.

The circadian pattern of temperature oscillation correlates with further neurologic signs (i.e., seizure in epileptic patients and intracranial hypertension in high-grade SAH) (1–3), and an elusive mechanism of this finding is that a normal circadian rhythm reflects normal hypothalamic neurogenesis (Figure 1) (1–6). Intriguingly, another potential marker of circadian rhythm, which could theoretically form a loop involving hCRYs and neurogenesis is blue light emission by the body. Endogenous blue light emission has been shown from human body (14). This emission displays circadian pattern and correlates with metabolism and reactive oxygen species (ROS) formation (14). Blue light stimulates CRY expression (11), and both—CRY (15) and ROS (16)—stimulate neurogenesis. Conversely, neurogenesis could close the loop by decreasing ROS formation, blue light emission, and CRY stimulation.

These observations raise the possibility that the circadian pattern of CRY expression correlates with circadian patterns of markers of inflammation and neurogenesis, neurologic signs in the following day–night cycle, and outcome in high-grade SAH.

## Hypothesis Testing

The primary analysis to test our hypothesis is the correlation of the ratio between the level of hCRYs in the cerebrospinal fluid (CSF) during the nocturnal and the preceding diurnal periods (CRY_n/d_) of acute phase of high-grade SAH patients with outcome after 1 year. This level can be determined by immune-enzymatic assay (ELISA). In addition, bearing in mind the relationship between CRYs and inflammation, the ratio can be calculated also regarding the expression of hCRYs in white blood cells (WBCC_n/d_) (and their different types, e.g., mononuclear cells).

Due to potential therapeutic implications of the hypothesis (as detailed below), a crucial analysis is the correlation between CRY_n/d_ and TV and ICP_24_. A caveat of this analysis is that TV has been shown to correlate with ICP_24_ only under strict control of temperature, with daily mean <35°C (2).

The complete study to test the hypothesis proposed here includes our published protocol to assess multimodality monitoring, inflammation, and neuroregeneration markers in CSF and blood of high-grade SAH patients aiming at revealing a prognostic biomarker (4). Because of our previous work, we deem worthwhile including in our original protocol principally the analysis (from blood and CSF samples of SAH patients) of markers related to circadian rhythms (melatonin), thermoregulation (RANK) (17), and hypothalamus- or hypophysis-produced hormones (growth hormone-releasing hormone, growth hormone, gonadotropin-releasing hormone, and oxytocin) (Table 1). Accordingly, the pineal gland and neurohypophysis are circumventricular organs, and the hormones mentioned above stimulate neurogenesis (18, 19).

**Table 1:** Parameters for correlation analysis with CRYn/d.

Parameter	Method	Function	Timing after night cry assessment (18:00)
**Molecular and cellular markers in blood or CSF**			

**WBCC**__n/d__	**qRT-PCR**	**Systemic circadian CRY oscillation**	−**12 and 0 h**
WBCC_n/d_ (mononuclear)	qRT-PCR	Systemic circadian CRY oscillation	−12 and 0 h
Blood and CSF, interleukin-6, TNF-α, and CRP	ELISA	Inflammation	12 and 24 h
Blood and CSF IL-10 and T_h_	ELISA and FACS	Anti-inflammatory	12 and 24 h
CSF MCP-1 and SDF-1	ELISA	Inflammation/neurogenesis	12 and 24 h
Blood and CSF histamine	ELISA	Neuroprotection/neurogenesis	12 and 24 h
CSF microglia and mast cells	FACS (CD-68 and CD-117/c-kit)	Inflammation/neurogenesis	12 and 24 h
CSF CD133+ cells	FACS	Neurogenesis	12 and 24 h
CSF ATP and ADP	ELISA	Redox state/purinergic pathway	−12 and 0 h
CSF cytochrome c and phosphoethanolamine	ELISA	Redox state/mitochondrial respiration	−12 and 0 h
CSF hydrogen sulfide	ELISA	Neuroprotection/neurogenesis	−12 and 0 h
CSF neuroglobin	ELISA	Oxygen metabolism	−12 and 0 h
Blood and CSF GH, GHRH, GnRH, oxytocin, vasopressin, and melatonin	ELISA	Circumventricular organ (median eminence, neurohypophysis, pineal gland) function	−12 and 0 h
CSF RANK	ELISA	Thermoregulation	−12 and 0 h

**ICU multimodality monitoring**			

**TV**	**Continuous core body T monitoring**	**ICP**__24__ **prediction**	−**12 to 12 h**
**ICP**__24__	**Continuous ICP monitoring**	**Intracranial hypertension**	**12 to 36 h**
Brain anatomy	CT scan	DCI	Clinical indication
P_ti_O_2_	Parenchymal probe	Brain hypoxia	12 to 36 h
Lactate/pyruvate	Microdialysis	Brain metabolism	12 to 36 h (3 h intervals)
MCA_v_, Lindegaard index, CO_2_ reactivity	Transcranial Doppler	Vasospasm/cerebral autoregulation	12 and 24 h
Alpha–delta ratio	Continuous EEG	DCI prediction	−12 to 12 h

**Outcome**			

**mRS**	**Clinical assessment**	**Functional**	Discharge, 1, 3, 6, and **12mo**
GOS	Clinical assessment	Functional	Discharge, 1, 3, 6, and 12mo
Barthel index	Clinical assessment	Functional	Discharge, 1, 3, 6, and 12mo
MMSE	Clinical assessment	Cognitive	Discharge, 1, 3, 6, and 12mo
MoCA	Clinical assessment	Cognitive	Discharge, 1, 3, 6, and 12mo
Sickness Impact Profile	Clinical assessment	Health-related QoL	Discharge, 1, 3, 6, and 12mo
Short-form 36	Clinical assessment	Health-related QoL	Discharge, 1, 3, 6, and 12mo

*CSF collection at 6:00 and 18:00 at minimum every 3 days for CRY_n/d_ determination when external ventricular drain is placed, until 14 days after bleeding. Minimum, maximum, and mean CRY_n/d_ for each patient should be compared with any of the remaining parameters to search for correlation. WBCC_n/d_ and mononuclear WBC CRY levels can be used as an alternative parameter for circadian CRY level. Main parameters are in bold. The remaining parameters are options that can be selected according to the goal of the protocol or resources of the service*.

*ADP, adenosine diphosphate; ATP, adenosine triphosphate; CRP, C-reactive protein; CRY, cryptochrome; CRY_n/d_, ratio between CRY level at night (18:00) and the preceding day (6:00) in CSF; CSF, cerebrospinal fluid; DCI, delayed cerebral ischemia; EEG, electroencephalogram; ELISA, enzyme-linked immunosorbent assay; FACS, fluorescent-activated cell sorting; GH, growth hormone; GHRH, growth hormone-releasing hormone; GnRH, gonadotropin-releasing hormone; GOS, Glasgow Outcome Scale; ICP, intracranial pressure; ICP_24_, mean ICP in the day following TV monitoring; ICU, intensive care unit; IL, interleukin; MCA_V_, middle cerebral artery velocity; MMSE, mini-mental status exam; MCP, monocyte chemoattractant protein; MoCA, Montreal cognitive assessment; mRS, modified Rankin scale; P_ti_O_2_, brain tissue oxygen pressure; RANK, receptor-activator of nuclear factor κ B; qRT-PCR, real-time polymerase chain reaction; QoL, quality of life; SDF, stromal cell-derived factor; T, temperature; T_h_, T helper; TNF, tumor necrosis factor; TV, temperature variability; WBCC_n/d_, ratio between CRY expression in white blood cells at night (18:00) and the preceding day (6:00)*.

Also bearing in mind its intriguing presence in the circumventricular organs [pineal gland (20)/habenular zone (21), choroid plexus (22), hypothalamus parenchyma (22)], it would be interesting to quantify through cell sorting mast cells in the CSF of the patients enrolled in this type of protocol. The potential neurogenic system arises from zones without blood–brain barrier, whose permeability is dynamic across the sleep–wake cycle (23). An indication that mast cells may contribute to dynamic features of blood–brain barrier is that they contain granules of histamine, which increases locally the permeability of blood–brain barrier (21, 22). Histamine also decreases inflammation *via* activation of H2 receptor and is involved in neuroprotection associated with preconditioning (24). Furthermore, histamine increases neural stem cell proliferation and favors cell fate toward neuronal differentiation *via* activation of neural stem cell H2 and H1 receptors, respectively (24). The presence of mast cells in circumventricular organs, action of histamine to increase blood–brain barrier permeability, decrease inflammation, protect neurons from secondary injury, and stimulate neurogenesis suggest that mast cells may play a role in endogenous mechanisms of neuroprotection and neuroregeneration.

## Expected Results

In decreasing order of importance, the expected results for the study protocol summarized above are:

- CRY_n/d_ correlates with SAH outcome after 1 year;
- CRY_n/d_ correlates with TV and ICP_24_;
- CRY_n/d_ correlates with CSF and/or blood biomarker(s) of inflammation or neuroregeneration.

Some remarks should be mentioned regarding hypothesis testing and expected results (Table 1).

Cerebrospinal fluid collection for CRY determination will be carried out at 6:00 and 18:00, because they are the times of extreme core body temperature values. This procedure will be performed when an external ventricular drain is placed, at least every 3 days, and until 14 days after bleeding. For each patient, we will test three manners to calculate CRY_n/d_: highest ratio, lowest ratio, and mean ratio of all daily CRY_n/d_ values. CRY level will be calculated as hCRY1, hCRY2, and hCRY1 plus hCRY2 levels.

The role of inflammation in stroke and particularly in SAH pathophysiology is not totally established (25). For example, tumor necrosis factor alpha correlates with neuron damage or protection depending on the membrane receptor on which it acts (4). Interleukin-6 (IL-6) shows a similar dual effect depending on microenvironment conditions (4). Likewise, microglia participates in inflammation in the acute phase of stroke model, but in a later phase contributes to migration and differentiation of newly formed immature neurons (i.e., neuroblasts) (4).

The multifactorial nature of SAH pathophysiology hampers the determination of correlation between outcome and inflammation (25). SAH and brain injury trigger clinical features that lead to systemic inflammation (26), which on its turn may cause brain damage through seizures, for example (26). However, some studies showed that serum level of inflammation markers such as C-reactive protein (CRP) (27) and IL-6 (28, 29) correlates with SAH outcome.

Cerebrospinal fluid composition could in theory reflect a more straightforward level of brain inflammation, but factors such as amount of bleeding and use of external ventricular drain may interfere on results. A stronger correlation in comparison with serum inflammation markers was demonstrated between CSF level of the inflammation-related marker high mobility group box-1 protein and SAH outcome after 3 months (30). This marker and CRP (27) also correlated with clinical (Hunt and Hess, World Federation of Neurological Societies, and Glasgow Coma scales) and radiological (Fisher) scales known to be prognostic factors in SAH.

The use of the ratio between CRY level during day and night is necessary to assess the role of circadian rhythm in SAH and is an approach that may contribute to overcome certain caveats mentioned above.

First, a higher CRY_n/d_ would result from more remarkable sequential alterations between increase and decrease CRY level collected, respectively, during night and day, supposedly reflecting a closer to normal circadian rhythm. The use of this proportion instead of absolute values may diminish the influence of amount of bleeding on results.

Second, because CRY expression is ubiquitous, the cell type that serves as source of CRY in the CSF cannot be determined in this study protocol. A low CRY_n/d_ may reflect low increase in CRY expression in white blood cells during night and, therefore, a lower anti-inflammatory activity. Additionally, a low CRY_n/d_ may reflect a lower number of hypothalamus neurons in the same way that the level of the hypothalamus-produced hormones oxytocin and vasopressin is lower in the CSF of SAH cases with poor outcome (31). An indication whether the level of CRY in the CSF is altered due to less intense increase of CRY expression in white blood cells or due to brain damage may be revealed from the analysis of WBCC_n/d_ (or alternatively of CRY expression in CSF leukocytes).

This study protocol intends to be a pilot study and the primary endpoint will be 1-year outcome defined according to modified Rankin scale as good (0–3) or poor (4–6). This option may yield a preliminary conclusion using Mann–Whitney *U*-test.

Complementary, we will analyze other functional scales, namely, Glasgow Outcome Scale and Barthel Index. Because half of the survivors after SAH displays good functional outcome measured by modified Rankin scale, but half of these patients shows neuropsychological alterations (32), we will explore this issue through the Mini-Mental Status Exam (33) and Montreal Cognitive Assessment (MoCA) (32, 34, 35). Moreover, SAH outcome studies have been taking into account analyses of health-related quality of life, which in our study will be evaluated through Sickness Impact Profile and Short-Form 36 (36, 37). Regarding timing, all outcome tests will be performed at discharge, and 1, 3, 6, and 12 months after bleeding.

## Potential Implications of the Hypothesis

The confirmation of the expected results would represent one more piece of evidence that circadian rhythms parallel endogenous mechanisms of brain plasticity and can be used to predict neurologic status (Figure 1). Regarding the study protocol we propose here, neurologic status corresponds to intracranial hypertension during the acute phase of SAH and to functional outcome in the long term.

This confirmation may underpin therapies guided by circadian rhythm modulation. A recent study showed that hCRY2 senses magnetic field (8). Magnetic field can be manipulated in clinical practice through transcranial magnetic stimulation, which interestingly increases neurogenesis (38). Perhaps transcranial magnetic stimulation improves neurologic outcome in a series of neuropsychiatric diseases through stimulation of hCRY expression and consequent neurogenesis.

Blue light stimulation is another clinical measurement that may modulate circadian rhythm. In this regard, flashing light improves Alzheimer’s disease in experimental model (39). The retina is a major structure where hCRYs are expressed (10). Blue light also acts on hCRY expression in peripheral tissues. For example, jaundice improvement in children with blue light phototherapy correlates with increase in hCRY expression in mononuclear white cells (11). Therefore, blue light interferes in the level of hCRYs through central and peripheral mechanisms, and this is the reason for which it is interesting to assess hCRY expression in CSF and white blood cells in high-grade SAH patients and the correlation of this expression with TV and ICP_24_. In clinical practice, monochromatic phototherapy could follow the protocol described by Chen et al. who demonstrated CRY increase during treatment of jaundice in neonates (11). For example, in the first day of treatment, 20 W cool fluorescent bulbs could be used from 18:00 to 6:00 of the next day to reach 500 µW/cm^2^ measured by a illuminance meter. This procedure is expected to increase CRY during night (which could be monitored by further CSF collections). In the following days, light exposure could be tailored aiming at reaching a CRY_n/d_ that further studies occasionally reveal to be associated with good outcome.

**Figure 2:**
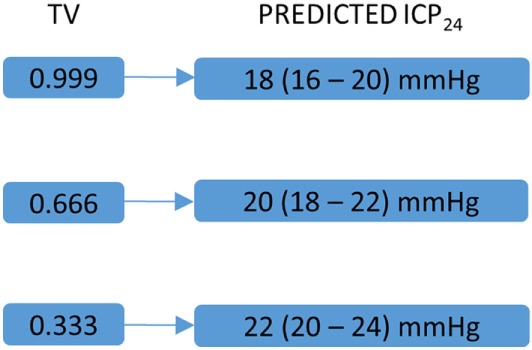
Key temperature variability (TV) values and corresponding predicted mean intracranial pressure in the following 24 h (ICP_24_). This figure is a schematic representation of key TV values and their corresponding predicted ICP_24_ [for detailed explanation, please see Ref. (2)]. Numbers between parentheses correspond to 80% prediction interval. These values could guide a further circadian rhythm modulation by stimulation of hCRY expression through transcranial magnetic and blue light stimulations.

**Figure 3:**
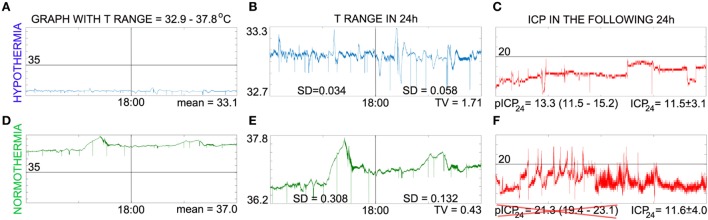
Caveat on intracranial pressure (ICP) prediction *via* temperature variability (TV) analysis. This panel illustrates that TV can predict ICP (in millimeter of mercury) only when mean daily T < 35°C and points to possible factor that contributes for this feature. **(A,D)** Show temperature in the same scale to visualize that absolute values were practically the same only during intravascular-induced hypothermia, which implicates that night–day ratio of coefficient of variation and night–day ratio of SD of temperature are practically the same only during hypothermia. In **(B,E)**, the temperature curves were zoomed in to show that temperature range is larger during normothermia. Note that the baseline changes in the second part of the day during normothermia [day is represented at the left side of vertical line of **(A,B,D,E)**], which reflects in the SD found; on the other hand, SD during hypothermia reflects principally temperature variation on a constant baseline. We are calculating the coefficient of variation of shorter periods (harmonics of 12 h) to see if the mean of these values can be used to derive a formula to predict ICP_24_. **(C,F)** shows the ICP curves of the next day. The predicted ICP (pICP_24_) matches ICP_24_ only during hypothermia. For details regarding calculation, please see Ref. (2), from which **(A–C)** were adapted; **(D–F)** have not been published previously.

If CRY_n/d_ or WBCC_n/d_ correlates with TV and ICP_24_ and transcranial magnetic stimulation and blue light interfere in the level of hCRYs, then the endpoint of these therapies in high-grade SAH could also be the value of CRY_n/d_ or WBCC_n/d_ that leads to a TV higher than 0.666 and consequently to a predicted ICP_24_ lower than 20 mmHg (Figure 2). Nonetheless, it is important to emphasize that these remarks are highly speculative and the practical use of these concepts depends on the confirmation of the hypothesis explained here, the replication of the study on ICP prediction by TV, and in the improvement of the TV analysis in such a way that it includes mean daily temperature values higher than 34.9°C (Figure 3). In our series, days with mean daily temperature higher than 34.9°C displayed higher temperature range. In these cases, the night–day ratio of coefficient of variation (SD/mean) was significantly different from the night–day ratio of SD, because mean night temperature over mean day temperature was not equal to one (contrarywise to what happens during hypothermia) (Figure 3). We are currently testing whether ICP_24_ can be predicted by TV during non-hypothermia periods using variables derived from the mean coefficient of variation of harmonics of 12 h obtained during day or night, similar to the formula to predict ICP_24_ already described (Figure 3).

## Conclusion

We have previously shown that the adult human brain harbors a potential neurogenic system whose integrity could be monitored by the analysis of circadian rhythms such as temperature oscillation. The pattern of circadian rhythms correlates with further brain injury, even under conditions in which the sleep–wake cycle is absent and the temperature is strictly controlled. This finding led us to postulate that light-independent mechanisms of circadian rhythm control may be involved in endogenous brain plasticity.

The circadian proteins CRYs display light-independent mechanism of circadian rhythm control, can be assessed in acute severe neurologic conditions, and their expression could theoretically be modulated by blue light and transcranial magnetic stimulation.

## Author Contributions

AN (first author): developed the hypothesis and wrote the paper. AN (second author): participated in previous work of the group on the paper subject, provided ideas, and revised the manuscript. JV and MT: participated in previous work of the group on this subject, provided ideas, and revised the manuscript.

## Conflict of Interest Statement

The authors declare that the research was conducted in the absence of any commercial or financial relationships that could be construed as a potential conflict of interest.
